# Photobioreactors for cultivation and synthesis: Specifications, challenges, and perspectives

**DOI:** 10.1002/elsc.202100070

**Published:** 2021-10-27

**Authors:** Santiago N. Chanquia, Guillem Vernet, Selin Kara

**Affiliations:** ^1^ Biocatalysis and Bioprocessing Group Department of Biological and Chemical Engineering Aarhus University Aarhus Denmark

**Keywords:** bioprocess engineering, photobiocatalysis, photobioreactors

## Abstract

Due to their versatility and the high biomass yield produced, cultivation of phototrophic organisms is an increasingly important field. In general, open ponds are chosen to do it because of economic reasons; however, this strategy has several drawbacks such as poor control of culture conditions and a considerable risk of contamination. On the other hand, photobioreactors are an attractive choice to perform cultivation of phototrophic organisms, many times in a large scale and an efficient way. Furthermore, photobioreactors are being increasingly used in bioprocesses to obtain valuable chemical products. In this review, we briefly describe different photobioreactor set‐ups, including some of the recent designs, and their characteristics. Additionally, we discuss the current challenges and advantages that each different type of photobioreactor presents, their applicability in biocatalysis and some modern modeling tools that can be applied to further enhance a certain process.

**Abbreviations**
LED: light emitting diode
PBR: photobioreactor
WLE: wireless light emitter

## 1 INTRODUCTION

**FIGURE 1:**
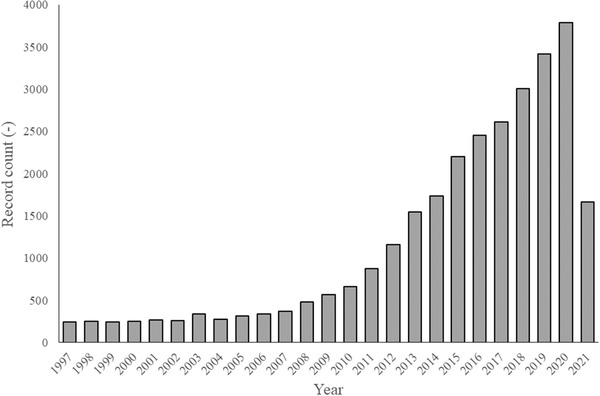
Number of microalgae publications published each year in the last 25 years, in which it is possible to see the growing interest in microalgae. Analysis made using “Web of Science” database searching “microalgae” as topic on the 15‐06‐2021

There are plenty of different species of phototrophic microorganisms, ubiquitous all around the globe, with estimations ranging from a moderate number of 30,000 to the astonishing count of 1 million [1]. These organisms are indeed so numerous, that it is calculated that they are responsible for almost 50% of the total photosynthesis of our planet [2]. Amongst them stand the microalgae and the Gram‐negative prokaryotic cyanobacteria. They stand out as mayor players for commercial applications, already being widely discussed for biofuel production [3], or as natural sources of valuable products such as pigments [4], bioactive compounds or bulk chemicals. Furthermore, they present exciting results for their application in the food and feed industry [5, 6]. An excellent example of this is the production of cyanobacteria of the genus *Spirulina*, frequently used for animal and human consumption because of their outstanding nutritional value [7, 8]. On the other hand, taking into account the alarming increase in global CO_2_ levels, which results in severe climate change effects, and considering the high sequestration capability that algae present, which is estimated in 1.83 kg of CO_2_ per kg of dry biomass [9], it was proposed that they could be part of the way towards a more sustainable economy [10]. Yet other environmental applications of algae in which they showed great promise is the treatment of affluents [11, 12], or as sustainable biofertilizers, which reduces the use of polluting synthetic fertilizers [11, 12, 13, 14, 15, 16]. All these applications have generated a growing interest in these organisms that is also reflected in the steady growth in the number of papers regarding this subject, as seen on Figure 1.

**PRACTICAL APPLICATION**

Photobioreactors are an attractive choice to perform microalgae cultivation and biocatalytic processes in an efficient way, sometimes even in a large‐scale. Although photobioreactors have been used for years, they are still being studied and improved using different shapes and illumination techniques in order to optimize the process.

In this review, we aim to gather the latest information about different photobioreactor set‐ups, including some of the most recent designs, their main characteristics, the main parameters affecting the photobioprocess, and the advantages and disadvantages of each type of photobioreactor in order to provide a clear idea of them, and acknowledge their present situation and future challenges. Furthermore, we discuss the application of photobioreactors in biocatalysis and the application of different mathematical modeling tools that can be used to improve the efficiency of a given process.

Since these microorganisms are photosynthetic, their growth is conditioned to the presence of a source of light. Nevertheless, this is not the sole factor to consider, since also pH, CO_2_, nitrogen availability, salinity, temperature, oxygen removal and medium mixing all play a major role, and must be tuned for each specific case, given that the growth rate and biomass maximum concentration of algae tend to vary between species [17].

One of the most common ways of growing phototrophic microorganisms is using open systems, such as raceway ponds. Although these are economic in terms of construction, operation and maintenance, the control on growth conditions tends to be poor, which makes them unsuitable for the production of fine chemicals, and pharmaceutical or food ingredients [9]. Additionally, there is a non‐negligible risk of contamination by parasites and predators of microalgae, such as rotifers or ciliates, which can destroy microalgal biomass in days, causing severe losses to productivity [17]. In contrast, closed photobioreactors (PBRs) offer an excellent control on culture conditions with minimal risk of contamination, but with a higher initial investment, and operational and maintenance costs [18].

The use of phototrophic organisms in biocatalysis has been a discipline in constant expansion in the last years, with several important fine chemicals being produced in this way both in industrial and lab scale [19], typically using either flat panel or tubular PBRs. Nevertheless, other types of PBRs have been the subject of many studies, with interesting advances regarding their design and application. We encourage the curious reader to take a look at some of the excellent works that have been published on the topic of PBRs, such as the ones from Carvalho et al. [20], Dasgupta et al. [21], Zitelli et al. [22], Chang et al. [23], and Płaczek et al. [24]. These authors present different perspectives on the topic and provide a great amount of information. Nevertheless, it should be considered that many years have passed since their publication, and therefore an update was needed. More recently, Johnson et al. [25], Sero et al. [26], and Legrand et al. [27] published very thorough reviews in which they briefly describe different types of PBRs, with an special emphasis in cultivation strategies in the first one, advances in biophotonics in the second one, and modelling in the third. Different from the abovementioned literature, in this review we significatively expand the information provided by these previous studies regarding the specifications of PBRs, while also providing examples of *state‐of‐the‐art* developments in the field. Furthermore, we also discuss the applicability of PBRs in biocatalysis, and provide a brief overview of mathematical modeling tools that might be applied in each process. With the objective of making this manuscript as practical as possible for the reader, the technical characteristics of PBRs and a comparison between different designs are presented in a tabulated form (Tables 1 and 2, respectively).

**TABLE 1:** Technical specifications of different PBRs

	PBR type		Temperature control	Mixing	Gas exchange
	Stirred tank		Heat exchanger/reactor jacket	Stirrer/impeller	Gas injection
Conventional	Tubular	Vertical column	Independent unit	Airlift/bubbles	Gas exchange at head space
		Horizontal tubular	Water spraying, shading	Circulation with pumps	Injection into feed. Dedicated degassing unit
	Flat panel		Heat exchange coils	Airlift/bubbles	Gas exchange at head space
	Bag		Independent unit	Circulation with pump	Injection in a separate unit
Unconventional	Pyramid		Independent unit	Airlift/bubbles	Gas injected through sparger
	Nature inspired		Thermostatic bath and shell and tube heat exchanger	Pump/small bubbles from turbulent flow	Gas injected through sparger
	Hybrid	Membrane	Independent unit	Circulation with pump/bubble mixing	Gas injection
		Open‐close	Heat exchanger	Recirculation with pumps	Gas injection in one of the units

**TABLE 2:** Comparison between different PBRs

	PBR type		Use in photo‐biotrans formations	Advantages	Limitations
Conventional	Conventional	Stirred tank	Yes [44]	Easier to control. Possibility of running axenic cultivation	Low surface to volume ratio, low efficiency of light absorption and low productivity [102]
	Tubular	Vertical column	Yes [55]	Good biomass growth, high efficiency of photosynthesis, high potential of scalability. Cheap and easy to maintain. Low energy use and suitability for algae immobilization	Small area of light exposition that is additionally reduced with the increase of column diameter. Low surface to volume area. Possibility of biofilm formation on reactors walls
		Horizontal tubular	Yes [17]	Large surface to volume ratio. Allow high biomass density	High energy consumption, photo bleaching, Scale up challenging due to the loss of the surface to volume ratio
	Flat panel		Yes [57]	Large surface of exposition to light. High surface to volume ratio. High productivity of biomass. Ability to maintain uniform access to light across entire volume of cultivation. Small concentration of dissolved oxygen	Increase of production scale requires the use of numerous modules [51]. Difficulties in control of cultivation temperature. Risk of fouling. Potential hydrodynamic stress in some algae species.Photoinhibition
	Bag		No	Good adaptability, simplicity, and cost‐effectiveness	Need for periodic bag replacement.Considerable amount of waste when using disposable plastic bags.Prone to photoinhibition and leakage
Unconventional	Pyramid		No	Small area of land needed. Completely automated, Possible to have internal and external illumination. Fully automated [26, 78]	Further studies are required
	Nature‐inspired		No	Extremely high surface area to volume ratio. Good performance in multiphasic flow	Further studies are required
	Hybrid	Membrane	Yes [88]	Easy operational maintenance and low operating temperature	High pressure drops, fouling formation and difficult to optimize in full scale plant
		Open‐close	Yes [86]	High surface area/volume ratio. Maximizes solar harvest	Need to improve the design's biomass and oil yields

## 2 PHOTOBIOREACTORS (PBRS)

Photobioreactors are defined, in most cases, as closed illuminated vessels in which the culture is not in direct contact with the environment, since the gas exchange with the atmosphere occurs through sterilized filters [17], and therefore the risk of contamination is significantly reduced. Additionally, this setup leads to less CO_2_ and water loss during the process, and productivities in general are much higher in comparison to an open system [28].

When designing a PBR, it would be ideal that it allows the cultivation of various species of algae, that the illumination is not fluctuating, uniform and reaches as much volume as possible inside the reactor. For obvious reasons, sunlight is the primary source of light for PBRs, but since their light intensity changes with the weather, the seasons, and depends on the latitude, other alternatives using artificial light sources have been explored, such as tungsten lamps, fluorescent lamps and light emitting diodes (LEDs). The first ones need large amount of energy to work, since they have an efficacy of around 15 lm/W, and just a small part of the emitted light is actually used by the cells, so fluorescent lights are a better choice. They are initially cheap and less energy consuming than the tungsten lamps, with a luminous efficacy between 35 and 100 lm/W, but tend to lose intensity over time. In these both cases, since the light is emitted in all directions, it is necessary to employ a system to direct the light into the PBR [29]. A more modern and efficient alternative is the use of LEDs, which are initially expensive but have a great lifetime and a good luminous efficacy, between 25 and 64 lm/W. Additionally, they emit in a narrow bandwidth that overlaps with the photosynthetically active radiation, and they can be built to focus the light at the PBR [30]. Despite all these great advantages, this technology is still not widely used in large scale cultivation given its high cost in comparison to solar light, but recently published articles show its potential for cultivation and biocatalytic purposes, in some cases adapting the LEDs to make internally illuminated PBRs [31, 32, 33, 34, 35, 36].

Another factor to consider is the fouling of the reactor due to the formation of films in the transmitting surfaces, which must be minimized since it significantly decreases productivity and sharply increases production costs. Unfortunately, there are not many studies regarding this subject, and the mechanisms of adhesion are still not fully understood, but nevertheless some recently published research articles propose interesting strategies to minimize it, such as the work of Fortunato et al. [37]. In this manuscript, the authors evaluate three physical fouling control strategies for the growth of *Chlorella vulgaris* in a membrane PBR: relaxation, backwash and nitrogen scouring. Relaxation aims to remove the foulant by stopping the permeate suction, which leads to the back‐diffusion induced detachment of the foulant agents by convective drag force, whereas the backwash consists in applying a permeate backflow through the membrane pores. In all cases the fouling rate was lower than the control, with a relative reduction in the fouling rate of 50% for backwash and relaxation and of 60% for nitrogen scouring. Another attractive recent contribution has been published by Talluri et al. [38], in which they concluded that PBRs built with hydrophilic materials, such as glass or poly(methyl methacrylate) are promising anti‐fouling surfaces for *Anabaena* sp. cultivation. To the reader further interested in fouling, the authors also recommend the review article by Zeriouh et al. [39], in which they analyze these phenomena in planktonic marine algae.

Lastly, it is also vital that high rates of mass transfer are achieved without damaging the cells nor hampering their growth, which also usually implies that the reactor needs to be able to work under intense foaming conditions [40]. This is especially important for large‐scale cultures and for shear‐sensitive microalgae, such as haptophytes, red algae, diatoms, and dinoflagellates. Although it is impossible to eliminate shear stress because mixing of the media is required for an efficient growing, it is desirable to reduce it. Regarding this topic, there is a nice review by Wang and Lan [41] in which they conclude that apparently air‐agitated PBRs are the most promising mixing systems for large‐scale cultivation at low shear stress.

**FIGURE 2:**
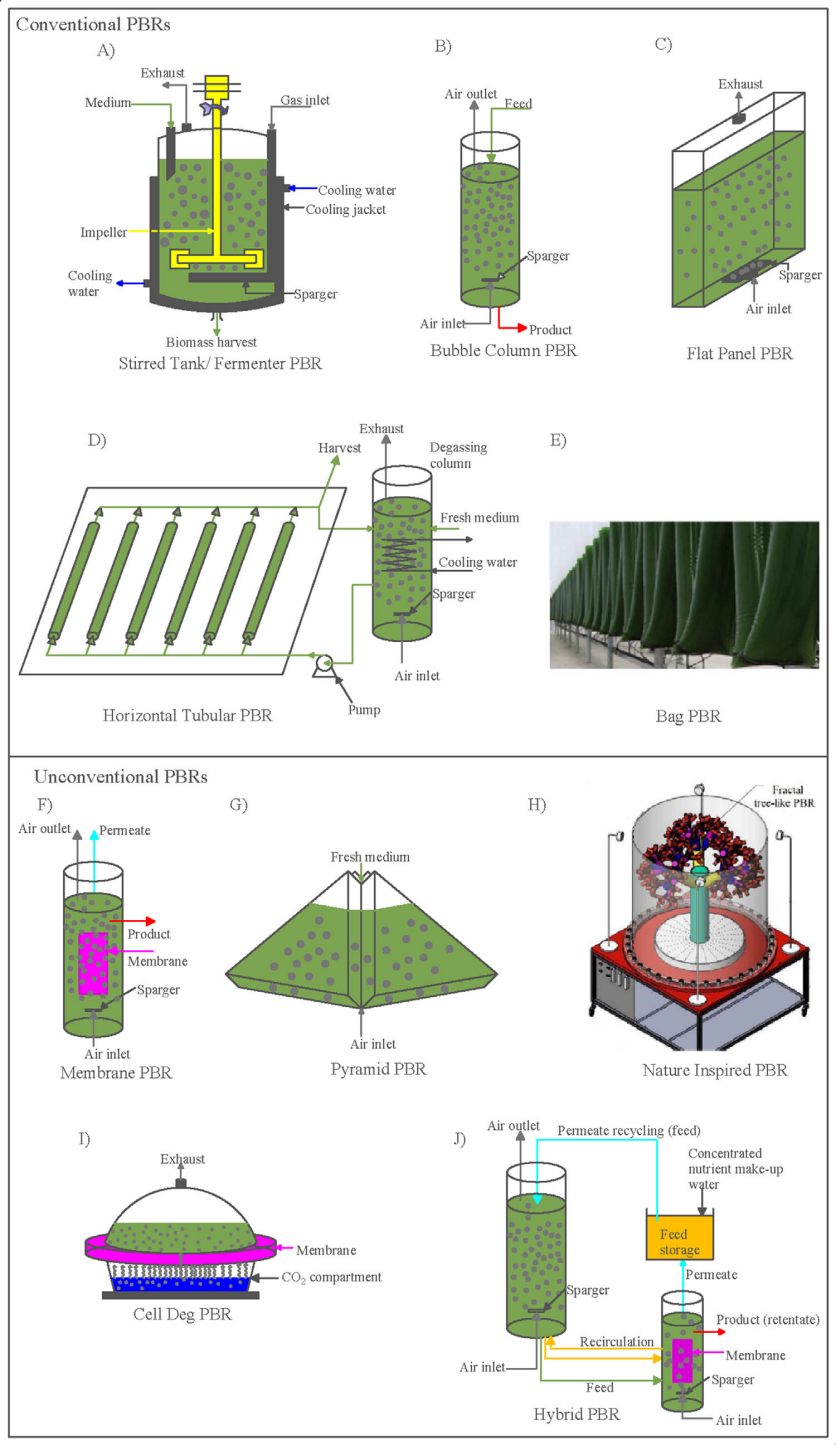
Different PBRs. (A) Stirred tank/fermenter, (B) Bubble column, (C) Flat panel, (D) Horizontal tubular, (E) Bag PBR, adapted from Sero et al. 2019 (F) Membrane PBR, (G) Pyramid, (H) Nature inspired, adapted from Zhao et al. 2021 I) CellDEG, J) Hybrid

As seen on Figure 2, there are several different types of PBRs and diverse ways of categorizing them. In this work, inspired by the work of Sero et al. [26], we decided to divide them in two groups, the conventional PBRs, which are the most traditional and widely‐used, and the so‐called unconventional PBRs, with a radical design and, so far, only operating at laboratory scale.

### 2.1 Conventional PBRs

#### 2.1.1 Stirred tank PBRs

In stirred tank PBRs, agitation is performed mechanically using one or more impellers, which may have different shapes and sizes, from lab scale until 250 L [20], with axial, radial, or mixed agitation [42]. Additionally, baffles can be included in order to reduce tangential agitation [43]. The main advantages of stirred tank photobioreactors are that they are able to ensure an efficient heat and mass transfer, alongside with a homogeneous nutrient dispersion when the stirring system is optimal [44]. Additionally, their simple design allows to easily modify stirred tank PBRs, in order to adapt them to be fit for many different specific processes and operation modes with good results [45].

The light capture capacity might be sometimes insufficient due to their low surface‐to‐volume ratio, which constitutes the main drawback of this type of photobioreactors, although it has been shown as a *proof‐of‐concept* that this can be improved using internal illumination [46]. Another disadvantage, in this case related to stirring, is that excessive mechanical agitation may cause high shear stress damaging the cell culture. Even though stirred tank PBRs are widely used for several processes in industry and in the laboratory because of their operational simplicity and successful results, they are not adequate for large‐scale photobioprocesses given their low surface to volume ratio, and the high electrical consumption needed to move the impellers [26, 44, 47, 48].

Continuous processes have attracted a great deal of attention in the last years and stirred tank PBRs have not escaped this trend, being used, for example, in a semi‐continuous fashion to treat an industrial effluent while producing carbohydrate‐rich biomass [49]. This allows to recycle industrial effluent waste while at the same time growing valuable biomass with recycled nutrients, which closes the process loop and is an example of circular process economy.

Another interesting option is to perform the process using a fed‐batch methodology, such as exemplified by the recent work of Ramanan and Rorrer [50], in which they study the growth of *Laminaria saccharina* adding nutrients in the aforementioned fashion. By applying this strategy, they observed that the productivity doubled in comparison to batch cultivation.

#### 2.1.2 Flat panel PBRs

Flat panel PBRs consist of two parallel, transparent flat panels on a frame, set to form a rectangular channel. This simple but witty geometry maximizes the illumination area per volume of the PBR, which results in a very efficient illumination of the cells [51]. Since the growth of phototrophic microorganisms depend, amongst other things, on the intensity and wavelength of the incident light, several research groups have applied different strategies to filter it, such as coloring the medium inside the PBR with dyes or applying different coatings on the panels [52, 53, 54].

When designing a flat panel PBR, it is important to take into account the panel alignment, which can be horizontal, vertical, V‐shaped, inclined or in an accordion arrangement [55]. In all cases the objective is to maximize photosynthetic efficiency, while also taking into account the equilibrium between it and the space needed to set up the PBR, and the possible photoinhibition, which actually is the main disadvantage of a flat panel in order to generate high amounts of biomass [51]. This big disadvantage explains why, even when the accumulation of dissolved oxygen is lower in comparison to, for example, tubular PBRs, the areal yields are usually lower [56]. An interesting modification to flat panel PBRs is to immobilize the cells on a grid, which enlarges the surface area of the reactor, as made by Wang et al. [57]. For further information about immobilized cell PBRs the authors recommend the recent work by Sagir et al. [58].

These PBRs do not use mechanical suspension of the cells, instead the mixing of the medium is performed by air bubbling, provided by a sparger found at the bottom. Although these PBRs are suitable for mass production of algae, they may not be the most profitable option for commercial applications due to the requirement of additional compartments and support materials when scaling‐up, which rises the cost significantly [51, 59]. However, flat panel PBRs can be used very efficiently for research or for the small‐scale production of phototrophic organisms. Nevertheless, challenges such as the difficulty in maintaining the temperature stable, the biofouling on the surface, the hydrodynamic stress lead to some algal strains, some degree of wall growth and incompatibility with some industrial fermentation equipment should be considered when using these PBRs [60, 61]. The most common way of upscaling these PBRs is to use multiple different, relatively small units, although flat panel PBRs up to 1000 L have been reported [62].

Regarding the different possible operation modes, it should be noted that operating in batch may limit microorganism growth because of nutrients depletion, or also because of excessive product accumulation which leads to inhibition [63, 64]. Hence, introducing continuously fresh media while removing the broth might solve at least some of these issues. From an industrial scale point of view, production in a continuously operated mode makes it possible to achieve between 2.5 and 5 times more efficiency when compared to a batch‐wise operated system [65].

In a recently published work [66], interesting parameters for a flat panel PBR flow system were studied, such as the effect of different residence times and light regimes for the production of a microalgal protein. The study concludes that the influence of residence time varies according to the biomass composition, having a higher effect in cyanobacterial strains than in green microalgae.

An interesting and original application for flat panel PBR has been proposed from the point of view of urban planning to develop a post‐COVID‐19 sustainable city [67]. The authors propose the use of semi‐continuous flat panel PBRs, arranged as a labyrinth, in public parks or technological gardens to produce biogas and absorb CO_2_, while also being a place for people's leisure.

#### 2.1.3 Tubular PBRs

Tubular photobioreactors are probably the most common, and there is a wide variety of them; they can be vertical, horizontal, near‐horizontal or in spiral/helical [26, 55, 60]. They have different characteristics and properties, but they are all made of a transparent material such as glass or plastic, and the aeration and mixing are done either with a pump or through an aeration system. On the other hand, given their large illuminated area they are amongst the most adequate PBRs for outdoor mass cultivation, although the helical and the near‐horizontal designs still have limited applicability at industrial scales [22].

Vertical designs consist of transparent vertical tubes that maximize light penetration [23, 55], and they are classified as either bubble‐column or airlift [26]. In the former, there are no other structures besides the sparger; therefore, the characteristics of the reactor depend entirely on the bubbling. Bubbles are formed by pumping air from the bottom of the column and, depending on the gas superficial velocity, the flow pattern changes. Below 1‐4 cm·s^–1^ the bubbles flow in a uniform fashion, resulting in a homogeneous flow, whereas over this velocity, the distribution of bubbles becomes uneven, which leads to local differences in density and results in an heterogeneous flow [68]. Amongst the main advantages of this design, we must mention its simplicity and low capital cost, which added to its good heat and mass transfer efficiency makes them very attractive for commercial uses [55].

Alternatively, the airlift column reactor consists of two linked compartments, called riser and downcomer. The medium circulation occurs via air supplied from the base of the column, which flows through the riser, while parallelly insufflating bubbles in the column. This leads to a circulation of the liquid in a defined direction, in which the “degassed” medium flows down through the downcomer, and the “gassed” medium ascends through the riser because of the density difference [68]. This flow pattern is one of the main strengths of this design, since it allows a very efficient mass transfer while also giving a “flashing” effect to the cells, since they pass continuously through dark (riser) and illuminated (downcomer) zones [69]. The main challenges that must be faced when using vertical tubular PBRs are related to the reflection of light at midday, and to the hydrodynamic and shear stress resulted from the nature of these PBRs: their vertical shape. This last effect is especially important if the columns are oversized [51].

Horizontal tubular PBRs probably are the most used amongst them all, having also many commercial applications due to their capacity to allow high biomass densities [17, 51]. They are made of transparent acrylic or vinylic polymer pipes, with a small internal diameter, maximum 60 mm [55], to maximize light penetration. To the best of our knowledge, the largest tubular PBR (with a vertical arrangement of the tubes) is located in Germany, with a capacity of 700 m^3^ and a total pipe length of 500 km. This impressive industrial plant is able to produce between 130 and 150 tonnes of dry mass (*Chlorella* sp.) per year [24].

This PBR arrangement has a large, illuminated surface‐to‐volume ratio while also preventing shading, with an especially great efficiency when the sun reaches its zenith, contrarily to vertical PBRs. Another advantage over vertical tubular PBRs is an improvement in the air residence time, which can provide more dissolved CO_2_ [17]. These PBRs can have many multiple orientations, but they all have the same basic principle, and in all cases the medium is mixed and circulates through the reactor thanks to an air pump. Even though horizontal tubular PBRs allow high culture densities, their upscaling poses a big challenge since they require a cooling system to avoid overheating [70], which size and cost increase with that of the PBR. Another complication is that these PBRs tend to present high levels of photoinhibition, given the accumulation of O_2_ and the high incident light intensity, which sometimes leads to lower productivities in comparison to vertical reactors [55].

Near‐horizontal PBRs are a variant of horizontal ones, in which the tubes are inclined at a certain angle, usually under 45°, while being supported by a frame [71]. The reason behind the inclination is that in this way the bubbles rise faster, resulting in better gas transfer coefficients [56], but this orientation also results in the rise of the bubbles generated by photosynthesis to the top of the PBR, causing light attenuation [26], which makes necessary to use an efficient mixing system, such as static mixers [72], to decrease bubble accumulation.

The helical design can have different shapes, and they all consist of a vertical structure with a flexible, transparent, small‐diameter tube rolled around, and a pump that allows medium circulation. This is a design that provides a uniform mixing and an efficient mass transfer of CO_2_ between phases. On the other hand, its two main disadvantages are that the tubes tend to clog because of excessive algal growth, and a significative difficulty in up‐scaling, since many geometrical parameters must be strictly defined, and also the energy consumption of the system increases sharply due of the need of stronger pumps, which in the end takes a toll in the global algaculture process cost [24, 26].

Diverse novel tubular designs are still being developed to improve the mass transfer and the surface to volume ratio. Lately, Cui et al. [73] developed a novel tangential double tube PBR (TDT PBR), which is composed of two horizontal tubes, one inside the other, where the inner tube has holes through which gas bubbles flow. Using computational fluid dynamics (CFD) simulations, they analyzed different arrangements and concluded that the best mixing and mass transfer coefficient were obtained when the inner tube was touching the lower side of the external tube, while using two rows of aeration holes. Applying this methodology, they accomplished a better transfer coefficient and decreased the shear rate, improving the microenvironment for microalgal cultivation. This is an important feature since, as shown by Le Gouic et al.[74], an increase in the mass transfer coefficient improves the performance of the PBR.

#### 2.1.4 Bag PBRs

Due to their low cost and sterility at the beginning of the cultivation, PBRs made of transparent plastic bags, usually polyethylene, are especially attractive for commercial applications [24, 71]. The bags are usually set to hang vertically in a parallel fashion, mounted on a frame, and the circulation of medium and aeration occurs thanks to a pump. The capacity of the bags is usually around 25 L, but bags up until 50 L have been reported [20, 24]. An interesting exception to this is the work by Zhu et al. [75], in which they use a plastic bag horizontally on a rocking platform to reduce energy cost.

The main advantages of plastic bag PBR design are its simplicity and cheap price [76]. Additionally, they present a good ratio between illuminated surface and total volume. Nevertheless, they present important limitations such as the need to replace the bags periodically, which for large operations poses an important environmental issue, poor mixing and, since the bags are fragile, there is a non‐negligible possibility that they might break and leak [26]. Additionally, it has been reported that an increase in the volume of the bags does not always lead to an increase in productivity [77], therefore the most common and efficient way to scale up a certain process using bag PBR is using several units, for which a large area is needed.

### 2.2 Unconventional PBRs

#### 2.2.1 Pyramid PBRs

Pyramid PBRs are one of the newest systems in algae cultivation [24]. They are characterized by their peculiar pyramid shape, made of acrylic, which allows adding external and/or internal illumination to obtain an efficient light capture while also maintaining a high intensity of light for optimum production rate. They are fully automated [26, 78], and the fluid is mixed using an airlift system. Arguably, the main advantage of pyramid photobioreactors is the small area required to install the photobioreactor in comparison to other PBRs [24, 26]. Unfortunately, these PBRs are still in experimental stage. Therefore, further studies must be done to achieve a better optimization, and to analyze the scalability and the economic viability associated with this design.

#### 2.2.2 Nature‐inspired PBRs

Nature processes can be considered, from a general point of view, continuously optimized for a long period of time [79], which makes them very attractive to emulate and apply for a certain process. As many times happened in history, for example with planes or submarines, several nature‐inspired researchers have proposed bold and interesting new PBR designs in the last years [80, 81, 82].

The purpose of the nature‐inspired PBRs is to increase the surface‐to‐volume ratio by imitating shapes found in nature [79, 83]. Also, the diffusion, heat and mass transfer are maximized using fractural structures based on the biological operations found in trees, lungs, birds, etc. [26]. The advances in 3D printing technology have allowed to manufacture a fractural new nature‐inspired photobioreactor, which imitates the bifurcations of a tree [79]. As the pyramid photobioreactor, they are still in experimental stage and further studies are needed to broaden the applicability of these PBRs [26]. In order to have a better understanding of the multiscale bubbles field and the light field interactions, numerical modelling and computational fluid dynamics (CFD) of the fractal tree‐like PBR have been performed [79]. The results obtained through them show that when the flow is optimized, the photosynthetic capacity and the microalgal activity are greatly enhanced given the synergistic effect with the light field.

Fibonacci, the famous mathematician, discovered centuries ago that the presence of logarithmic spirals is something very common in nature. This particular arrangement has been recently applied to PBR design by Díaz et al. [84, 85]. This new PBR gather the advantages of tubular PBRs, while improving the geometry using a helical spiral arrangement to imitate nature and obtain a higher surface to volume ratio with a capacity up to 1250 L. The results obtained confirm the improvement in light absorbance in comparison to a regular horizontal tubular, even though further optimizations and studies are needed.

#### 2.2.3 Hybrid PBRs

The hybrid photobioreactors combine different systems, such as open and close configurations, or membrane set‐ups, in order to obtain an optimal set‐up capable of achieving high biomass, good oil production yields, lower energy consumption, and economic sustainability [26, 86].

In the first case, the culture is exposed to an initial closed PBR to allow maximum production without contamination. When the desired biomass concentration is produced, the microalgae are exposed to harsh conditions in an open system to obtain the desired metabolites and lipid products [26, 86]. Recirculation is possible since both systems are connected and the medium pumped through them, therefore illumination and gaseous feed improves in comparison to a simple open system. Despite all the theoretical advantages, the presence of the open system is still a major limitation for the scalability of these systems, and also to achieve maximum algae growth, given their intrinsic high risk of contamination [26].

On the other hand, in porous membrane PBRs the medium is pumped to pass through the pores to the exterior part of the membrane, where the algae grow, since there the light intensity is the highest [25]. The main advantage of this type of PBR is its excellent gas dispersion, due to the great interfacial area provided by the membrane, but on the other hand these systems are highly permeable to water vapor [87].

Amongst membrane PBRs it is interesting to mention CellDEG, which is a PBR that consists on two‐tier vessels over a shaken platform. The inferior vessel is filled with a CO_2_ buffer, and it is separated from the upper vessel, where the culture grows, by a porous polypropylene membrane [88]. In this way, there is an steady supply of CO_2_ to the culture while avoiding high concentrations of O_2_. Using this setup, which is commercially available, a high density of biomass can be achieved, as shown in the work of Lindberg et al. [89, 90], but still the total amount of biomass that is possible to obtain is limited.

## 3 IN‐VITRO APPLICATIONS OF PBRS FOR PHOTOBIOCATALYSIS

Phototrophic organisms can be applied in combination with redox processes, using both natural and synthetic substrates [19]. This is due to the fact that biotransformations in microalgae are highly selective, have good atom economy, and also allow to design systems for cofactor regeneration [91], therefore it is tempting to combine their photosynthetic electron transport chain with an oxidoreductase [92, 93].

When synthesizing fine chemicals via biotransformation it is of paramount importance to maximize the productivity to make the process economically reasonable, especially at large scale. It is important to use the optimal organism, and to optimize the reaction parameters, particularly those regarding culture's exposure to light, since drawbacks such as self‐shading or poor light penetration have a great influence on the outcome of the process. One possible solution to these issues is the use of wireless light emitters (WLEs) [46, 94], that consist on a LED with a system that allows contactless power transfer via resonant inductive coupling. These WLEs are suspended inside the medium, therefore the light pathways get significantly shortened and the light reaches zones that otherwise would be dark [95]. Lately, this strategy has been successfully applied by Duong et al. for the production of biodiesel using a recently discovered fatty acid photodecarboxylase [96].

WLEs have been recently used together with cells of the cyanobacterium *Synechocystis* sp. PCC 6803 expressing the gene of the ene‐reductase YqjM for the reduction of 2‐methylmaleimide to (*R*)‐2‐methylsuccinimide with high optical purity (>99% *ee*). Compared to external source of light, illumination by floating wireless light emitters allowed a more than two‐fold rate increase. Under optimized conditions, 650 mg isolated enantiopure product (73% yield) was obtained. The results demonstrate the principle of internal illumination as a mean to overcome the intrinsic cell density limitation of cyanobacterial biotransformations, obtaining high reaction rates in a scalable photobioreactor [97].

## 4 MODELING OF PBRS

PBRs are considered to be comprised of four phases, the algae cells form a solid phase, the liquid growth medium, a gaseous phase containing CO_2_ and O_2_ and the light‐radiation field [83]. Considering the complexity of these systems it is of interest to develop mathematical expressions to model their behavior. The growth models most widely used are those of Monod and Andrews‐Haldane [27], both of which use experimental data to perform predictions, but do not explain nor analyze the phenomena behind the system behavior. These models are similar, the difference being that the latter introduces a term for substrate inhibition. More novel approaches profit from the advancements in artificial intelligence using deep‐learning techniques to find the best parameters for algae growth [98].

On the other hand, predictive and semi‐predictive models are based on the analysis of the parameters that control growth, such as algal metabolism and photosynthesis, and therefore need a great amount of theoretical knowledge [27].

It is noteworthy that, when all the other parameters are optimized, the process efficiency depends solely on the light absorption efficiency which, for several strains, depends lineally on the pigment content of the microalgae [99], therefore each microalgae must be modelled differently, being the optimal conditions those that minimize the occurrence of shaded zones, which will consequently affect the PBR design [100]. To further delve into details the authors highly recommend to the curious reader to read through the reviews of Gao et al. [101] and Legrand et al. [27].

## 5 CONCLUDING REMARKS

In a world in which the consumption of energy is unceasingly rising, and where most of this energy comes from non‐renewable sources, fuels and other products sustainably obtained must play a global major role towards a greener future. In this context, algae present themselves as a very attractive tool to fight climate change, and achieve the goals proposed in the Paris Agreement. There are still many challenges to face when using PBRs (Table 2), especially regarding industrial scale production of biomass, while also optimizing energy consumption and light harvesting, but we firmly believe that these setbacks are as challenging as they are rewarding. Combining the great variety of PBR types (Table 1) together with technologies such as light filters, different types of illumination and/or novel aeration or mixing methods is likely to result in new efficient strategies for large scale cultivation of biomass and production of useful substrates.

## CONFLICT OF INTEREST

The authors declare no conflict of interest.

## Data Availability

Data sharing not applicable – no new data generated.
